# SARS-CoV-2 reinfection in patients negative for immunoglobulin G following recovery from COVID-19

**DOI:** 10.1016/j.nmni.2021.100926

**Published:** 2021-08-02

**Authors:** A.M. Ali, K.M. Ali, M.H. Fatah, H.M. Tawfeeq, H.M. Rostam

**Affiliations:** 1) Department of Chemistry, College of Science, University of Garmian, Kalar, Kurdistan Region, Iraq; 2) COVID-19 Laboratory, Qala Hospital, Garmian General Directorate of Health, Ministry of Health, Kalar, Kurdistan Region, Iraq; 3) Medical Lab Technology Department, Kalar Technical College, Sulaimani Polytechnic University, Kalar, Kurdistan Region, Iraq; 4) Immunology & Immuno-bioengineering Group, School of Life Sciences, Faculty of Medicine & Health Sciences, University of Nottingham, Nottingham, NG7 2RD, UK; 5) College of Medicine, University of Garmian, Kalar, Kurdistan Region, Iraq

**Keywords:** COVID-19, immunoglobulin G, reinfection, SARS-CoV-2

## Abstract

While many patients infected by severe acute respiratory syndrome coronavirus 2 (SARS-CoV-2) eventually produce neutralising antibodies, the degree of susceptibility of previously infected individuals to reinfection by SARS-CoV-2 is currently unknown. To better understand the impact of the immunoglobulin (IgG) level on reinfection in recovered coronavirus disease 2019 (COVID-19) patients, anti-nucleocapsid IgG levels against SARS-CoV-2 were measured in 829 patients with a previously confirmed infection just after their recovery. Notably, 87 of these patients had no detectable IgG concentration. While there was just one case of asymptomatic reinfection 4.5 months after the initial recovery amongst patients with detectable anti-nucleocapsid IgG levels, 25 of the 87 patients negative for anti-nucleocapsid IgG were reinfected within one to three months after their first infection. Therefore, patients who recover from COVID-19 with no detectable anti-nucleocapsid IgG concentration appear to remain more susceptible to reinfection by SARS-CoV-2, with no apparent immunity. Also, although our results suggest the chance is lower, the possibility for recovered patients with positive anti-nucleocapsid IgG findings to be reinfected similarly exists.

## Introduction

Coronavirus disease 2019 (COVID-19) is an infectious disease caused by a 2019 novel coronavirus 2019-nCoV [1], severe acute respiratory syndrome coronavirus 2 (SARS-CoV-2), which was named so given the similarity of its symptoms to those induced by severe acute respiratory syndrome [2]. Since the first reports of viral pneumonia of unknown origin emerged from China in late 2019, this disease has spread across the world, with new cases reported daily. The clinical manifestations of COVID-19 range widely from asymptomatic to mild, moderate and rapidly progressive severe (pneumonia) disease that can lead to death in some individuals [[3], [4], [5]]. The moderate clinical symptoms of patients with COVID-19 include fever, dyspnoea, fatigue, dry cough, myalgia and pneumonia. In severe cases, affected patients may experience acute respiratory failure, septic shock and organ failure that might culminate in death [6,7].

Transmission of SARS-CoV-2 from infected people to others is suggested based on epidemiology and clinical evidence [8,9], with even asymptomatic infected individuals suggested of being capable of transmitting the virus [10,11].

Infection by SARS-CoV-2 leads to a detectable immune response, but the susceptibility of previously infected individuals to reinfection by SARS-CoV-2 is not well understood given the brevity of the worldwide pandemic to date. Generally, infection results in the generation of neutralising antibodies in patients [12,13]. SARS-CoV-2 has the capacity to escape innate immune responses, which allows the pathogen to produce large numbers of copies in primarily infected tissues, usually airway epithelia [14]. Principally, patients who recover from infectious diseases such as influenza A virus are usually immunised henceforth against infection by the causative virus for a period of time; however, reinfection by respiratory viruses is extremely common among humans of all ages due to these viruses’ progressive evolution through RNA genome mutations that lead to antigenic drift and immune escape. However, the complete mechanisms governing our susceptibility to recurrent viral infections remain poorly understood [15,16]. Although some studies indicate the persistence of protective immunoglobulin IgG levels in the blood, saliva and other body fluids for months after infection with SARS-CoV-2 [17,18], limited numbers of case studies of patients with COVID-19 have reported positive test results after the disease symptoms had resolved and negative test results were recorded, supporting the possibility of reinfection [[19], [20], [21]]. These reports included both patients with mild disease [22,23] and others with more severe conditions [21,24].

This study aimed to report an additional group of COVID-19 patients who were reinfected by SARS-CoV-2 and argue that the IgG level is a potential marker of the reinfection risk.

## Materials and methods

### Study population

A prospective follow-up study included a group of 829 patients admitted to Qala Hospital, Kalar, Kurdistan region, Iraq, from the last week of May until the middle of October 2020.

### Real-time reverse-transcription polymerase chain reaction (RT-PCR) assay for the diagnosis of SARS-CoV-2

Pharyngeal swabs were administered to extract SARS-CoV-2 RNA from each patient; then, the total RNA was extracted using the AddPrep Viral Nucleic Acid Extraction Kit (Addbio Inc., Daejeon, South Korea). Next, the presence of the SARS-CoV-2 virus was detected by real-time RT-PCR amplification of the SARS-CoV-2 open reading frame 1ab (ORF1ab) and envelope (E) gene fragments. The amplification reactions were carried out with 10 μL of 2X RT-PCR master mix, 5 μL of primer/probe mix and 5 μL of template RNA for a final volume of 20 μL using the PowerChek SARS-CoV-2 Real-time PCR Kit (Kogenebiotech, Seoul, Korea), described previously [25]. We followed the kit’s instructions and adopted the following thermocycler protocol: 50°C for 30 minutes and 95°C for 10 minutes, followed by 40 cycles of 95°C for 15 seconds and 60°C for one minute. Positive and negative controls for both genes (ORF1ab, E) were used in each run according to the kit’s instructions. When findings regarding the two target genes (ORF1ab, E) were positive according to specific real-time RT-PCR, a sample was defined as positive if the viral genome was detected at the cycle threshold value (Ct-value) of 36.7 or less (initial infection), while the Ct-value of greater 36.7 was defined as indicating a negative test result or recovery (i.e., the disappearance of signs and symptoms in a previously RT-PCR positive patient). Symptomatic persons with the second positive RT-PCR test were considered as reinfected patients.

### Enzyme-linked immunosorbent assay

A serum sample was collected from patients with a confirmed SARS-CoV-2 RT-PCR test result just after their recovery with a while of 12.23 ± 2.3 days (recovery time average).

The anti-nucleocapsid IgG antibody level was assessed using a commercially available SARS-CoV-2 IgG test kit (Pishtaz Teb Diagnostics, Tehran, Iran) targeting the IgG antibody against the nucleocapsid (N) antigen of the SARS-CoV-2 virus. Sera were diluted 1:101. First, 10 μL of the specimen together with 1000 μL of the sample diluent was processed in a 96-well test kit. Then, 100 μL of each control serum (without dilution) and diluted specimens were placed into the appropriate well, with the first two wells chosen as blanks and the next two chosen as negative control wells. Positive controls were used as duplicates, and the other wells were used for samples. Based on the manufacturer’s formula, the following cut-offs were applied: 1.1, positive; 0.9 to 1.1, equivocal; and less than 0.9, negative.

### Ethics declarations

All methods were used in accordance with relevant guidelines and regulations. Also, we confirmed that all experimental protocols were approved by the ministry of health of KRG (no. 14891 on 22/12/2020) and the Ethics Licensing Committee at Kalar Technical Institute/Sulaimani Polytechnic University (no. 02 on 01/08/2020). In addition, informed consent was obtained from all study participants or a parent and/or legal guardian if the individual was younger than 18 years of age.

## Results

**Fig. 1:**
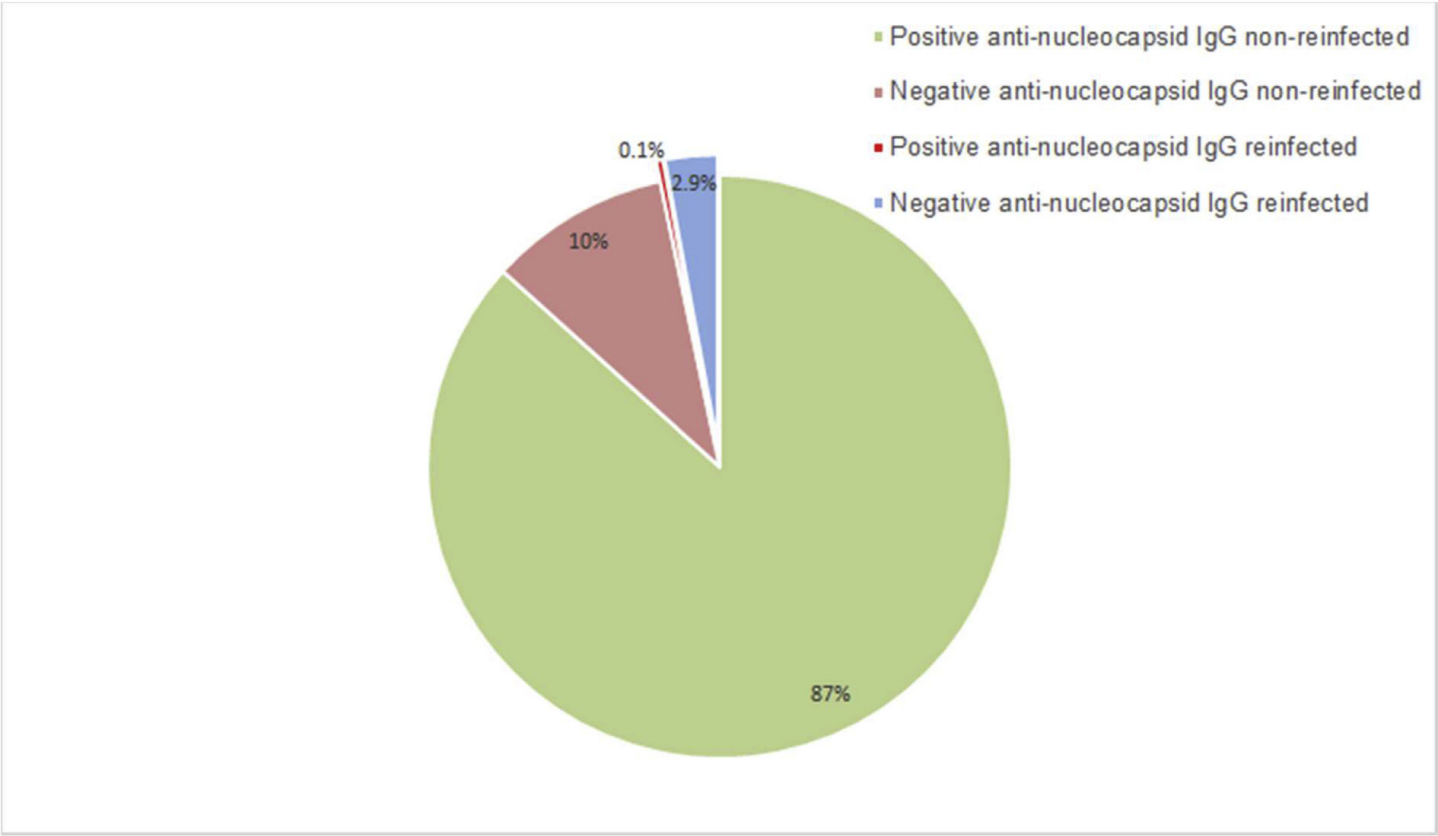
Patients with COVID-19. Among a total of 829 patients with COVID-19, 86 (13%) showed negative findings for anti-nucleocapsid IgG specific to SARS-CoV-2. Twenty-five (2.9%) patients were reinfected during the study period, while 61 (7%) patients remained healthy. A single patient with anti-nucleocapsid IgG positivity was reinfected (0.1 %).

Our study found that 86 patients tested negative for IgG specific to SARS-CoV-2 after recovery among a population of 829 patients who were infected with SARS-CoV-2 for the first time. Twenty-six patients (14 male and 12 female patients, aged 10–60 years old) were reinfected after recovery with the rate of 3.13%; of these, 25 patients were in the IgG-negative group, and only one patient was IgG-positive (Fig. 1).

Just during the first week after recovery, anti-nucleocapsid IgG antibodies against the SARS-CoV-2 were found in 96.2% of the serum of the reinfected patients. Only one patient was reinfected, although the anti-nucleocapsid IgG result remained positive after recovery from COVID-19 (Table 1). Most IgG-negative patients presented with just a couple of signs of COVID-19, including fever (96%) and myalgia (68%) and continued cough (<15% cases), while reinfected patients suffered more signs, including fever (96%), myalgia (88%), continuous cough (88%), anosmia and ageusia together (72%) (Table 2). In addition, after reinfection, more than 95% of the reinfected COVID-19 seroconvert patients had been protected as evidenced by anti-nucleocapsid IgG antibody induction. Surprisingly, there was no detectable IgG concentration in a male patient who had most of the common signs and symptoms of COVID-19 during both his first infection and reinfection. Also, an immunocompetent male patient (no. 26 in Table 1, Table 2) showed a serum IgG level of 5.87 s/ca against SARS-CoV-2 nucleocapsid after recovery but was reinfected 138 days later. Interestingly, the reinfection induced his immune system to produce IgG level by an amount (2.08 s/ca) less than the first infection. The occurrence of reinfection in the group ranged from 26 to 138 days after recovery from the initial infection (Table 1).

**Table 1:** COVID-19 data of the 26 reinfected patients in this study. The average Ct value for patients with anti-nucleocapsid IgG negative, 31.6 and for anti-nucleocapsid IgG positive, 21.3

No.	Gender	Age (years)	Infected COVID-19 patients			Recovery time period after disease confirmation	Reinfected COVID-19 patients		
			Date of positive RT-PCR result for SARS-CoV-2 infection	Ct Values	Anti-nucleocapsid IgG (s/ca) after recovery		Reinfection after (days of) recovery	Ct values	Anti-nucleocapsid IgG (s/ca) after Recovery
1	M	22	Jul	32.44	Negative	10	89	22.43	6.7
2	F	34	Aug	31.52	Negative	17	55	19.81	10.3
3	M	27	Sep	30.12	Negative	11	26	21.52	7.3
4	F	14	Aug	29.89	Negative	10	37	16.74	9.3
5	M	48	Aug	31.77	Negative	13	55	23.26	15.5
6	F	45	Sep	35.07	Negative	9	39	17.95	10.7
7	F	41	Aug	30.01	Negative	14	42	29.43	11.3
8	M	50	Aug	33.81	Negative	12	46	11.94	10.3
9	F	55	Aug	32.36	Negative	11	53	17.12	5.35
10	F	45	Aug	34.09	Negative	13	35	21.46	11.2
11	M	49	Jul	29.98	Negative	9	76	17.44	7.22
12	F	47	Aug	33.43	Negative	9	45	22.5	11.2
13	M	41	Aug	27.71	Negative	15	34	15.65	7.4
14	M	39	Aug	30.36	Negative	14	50	20.23	12.51
15	F	42	Aug	31.22	Negative	15	42	18.27	11.5
16	M	46	Aug	34.09	Negative	12	62	16.89	7.11
17	F	41	Aug	30.33	Negative	10	49	21.33	8.37
18	M	45	Jul	31.21	Negative	13	72	20.32	5.11
19	F	37	Aug	33.87	Negative	14	40	26.11	10.3
20	M	38	Aug	28.87	Negative	12	59	29.31	6.3
21	M	43	Aug	33.31	Negative	17	42	31.11	Negative
22	M	50	Aug	29.47	Negative	13	53	19.74	9.3
23	M	26	Aug	30.82	Negative	10	49	27.12	7.25
24	F	43	Aug	29.91	Negative	13	52	16.73	6.21
25	F	24	Aug	34.55	Negative	10	54	26.17	11.9
26	M	39	May	28.03	5.87	12	138	23.12	2.08

**Table 2:**
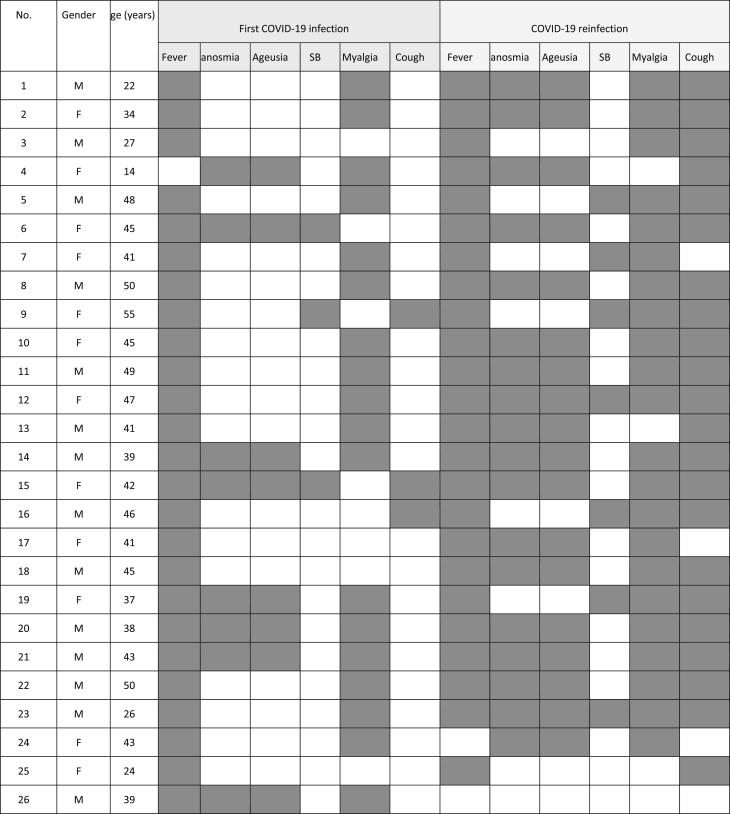
Sign and symptoms among all reinfected patients during both infection and reinfection, anosmia, ageusia; shortness of breath, SB; dark area, positive; light area, negative

## Discussion

Approximately 90% of recovered COVID-19 patients produce a detectable level of IgG [25]. In our study, among 829 infected cases, 742 IgG-positive (against SARS-CoV-2 nucleocapsid) recovered patients were identified. Studies demonstrated that detection of nucleocapsid antibody against SARS-CoV-2 is more sensitive than antibody towards spike protein in COVID-19 patients; Burbelo et al., concluded that the sensitivity and specificity of nucleocapsid protein antibodies peaks to 100% in SARS-CoV-2 when compared to spike protein antibodies that reached 91% and 100%, respectively [26]. Similarly, Brochot et al., confirmed that the detection of specific antibodies against SARS-CoV-2 nucleocapsid was more sensitive than S1 or S2 subunits [27]. A study that included 222 patients conducted by Zhang et al., concluded that anti-nucleocapsid antibodies were detected 4 days after onset of symptoms [28]. However, other studies suggested a 10 days incubation period for anti-nucleocapsid IgG production [29]. Thus, the suggested enough period of time for the immunoglobulin production in all patients was considered in the current study. Studies indicate that the viral load plays a crucial role in inducing the immune system to produce an adequate IgG level [[30], [31], [32]], which can be an indicator of the severity of the disease [33]. Confirming that, we found negative anti-nucleocapsid IgG patients with lower viral load (Ct value mean of 31.6 ± 2.1), while viral load in positive anti-nucleocapsid IgG reinfected patients was higher (Ct value mean of 21.3 ± 4.7). Therefore, it may be postulated that those patients who recovered from SARS-CoV-2 infection with IgG negativity in this study were exposed to a lesser amount of viral antigen.

The reinfection rate in the present study was greater than those recorded by Hall et al. [34] and Graham et al. [35]. SARS-CoV-2 genomic sequences from the reinfection cases in the study area have not been concluded yet, which raises the expectation of new phylogenetically distinct variants of SARS-CoV-2 that makes them more virulent.

The degree of protective immunity conferred by prior infection and the possibility of reinfection by SARS-CoV-2 is not well understood [21]. This study reports that around 10% of recovered COVID-19 patients showed no detectable anti-nucleocapsid IgG concentration after recovery and prior to their reinfection. A pair of studies from Hong Kong and Ecuador have also reported SARS-CoV-2 reinfection in two anti-nucleocapsid IgG-negative patients who previously recovered from COVID-19 [22,23]. The anti-nucleocapsid IgG level appears to be associated with the severity of the illness during the first infection; studies have shown that patients with mild symptoms had no/lower antibody titers as compared with those patients with more severe symptoms [22,33,36]. In our study, the degree of disease severity in the reinfection period was worse in most patients than during the first instance of COVID-19. A similar case study was reported in a 46-year-old male Ecuadorian patient [24]. This contradicts the findings of a couple of case studies in which patients were asymptomatic during their reinfection period but were symptomatic during their first infection [22,23]. The increase in disease severity in reinfected patients can be due to a high viral load or a change in virus virulence, which might have facilitated reinfection [21,37]. The lack of a detectable level of IgG against SARS-CoV-2 nucleocapsid during the infection period in mild or asymptomatic patients possibly makes them more susceptible to reinfection. Therefore, in the current study, it has been observed that the vast majority of patients who showed detectable levels of anti-nucleocapsid IgG after COVID-19 were thus mostly protected from reinfection, although the time period of the immunity conferred by IgG against SARS-CoV-2 nucleocapsid has not been concluded yet [22]. Unexpectedly, a male patient was reinfected with COVID-19 without inducing anti-nucleocapsid IgG production a second time, which raises the question of possible reinfection for a third time. Also, another male patient had detectable amounts of IgG during his first infection and was reinfected after 138 days with no symptoms. That may be due to a decrease in his anti-nucleocapsid IgG level as time passed, as studies have suggested neutralizing IgG levels start to decrease at six to 13 weeks after infection resolution [33,[38], [39], [40], [41], [42]]. Mysteriously, the reinfection in the aforementioned patient induced a low level of a detectable serum IgG concentration, which, most likely due to the low level of induced immunoglobulin against the virus [43,44]. This raises questions concerning the presence of adaptive immunity in COVID-19 reinfected patients.

## Conclusion

To conclude, a lack of anti-nucleocapsid IgG in patients who have recovered from COVID-19 may lead some to become infected. Anti-nucleocapsid IgG production possibly indicates the severity of the signs and symptoms of COVID-19. Further studies are needed to consider the efficiency and sustainability of IgG, which are likely to play a vital role in the success of the COVID-19 vaccine industry.

## Author contributions

AMA performed lab work. KMA, MHF, HMT and HMR contributed to writing and preparing the manuscript equally.

## Transperancy declaration

We are authors of the article titled ‘Severe Acute Respiratory Syndrome Coronavirus 2 Reinfection in Patients Negative for Immunoglobulin G Following Recovery from Coronavirus Disease 2019’. We confirm that we do not have any conflicts of interest to report for the submitted manuscript. No funding covered the work.

## Abbreviations

COVID-19: Coronavirus disease 2019
SARS-CoV-2: Severe acute respiratory syndrome coronavirus 2
IgG: Immunoglobulin G
RT-PCR: Real-time reverse-transcription polymerase chain reaction
RNA: Ribonucleic acid
ORF1ab: Open reading frame 1ab gene
E gene: Envelope gene
KRG: Kurdistan Region Government
